# Supplementary material for: Supporting Traumatic Grief: A Qualitative Analysis of Helper’s Lived Experience
Source: Int J Environ Res Public Health. 2022 Nov 30;19(23):16002. doi: 10.3390/ijerph192316002 (PMC9738878; doi:10.3390/ijerph192316002)
Supplement: Supplementary file 1 [file ijerph-19-16002-s001.zip › ijerph-2020042-supplementary.pdf]

Supplementary Materials

Table S1. Ad hoc questionnaire.

1. Experiences of traumatic grief: Yes; No
2. You are currently: operator; ex-operator
3. For how long you have been or were an operator of the De Leo Fund? Zero to 6 months; from 6 to 12 months; for more than 12 months?
4. How often do you do/have done shifts on the toll-free number of the De Leo Fund? 1-2 per month; 3-5 per month 5 or more shifts per month?
5. Before becoming an operator for the De Leo Fund, what expectations did you have regarding this role?
6. What made you want to become a helper in the area of traumatic bereavement?
7. In your opinion, what are the most critical aspects of your experience as an operator?
8. Do you feel that your experience as an operator at the De Leo Fund has/has had an effect in your personal relationships? If so, how?
9. Before starting to be an operator, did you consider the possible emotional impact that the job could have on you?
10. What feelings and emotions did you have when you came in contact with survivors of traumatic bereavement?
11. How did you try to manage the feelings and emotions you experienced when interacting with survivors of traumatic bereavement?
12. Being an operator of De Leo Fund has changed you in any way? If so, how?
13. How useful do/did you find the training received in managing the emotional impact with survivors of traumatic bereavement?
14. What kind of support offered by De Leo Fund did you find as most useful in managing your interaction with survivors of traumatic bereavement?
15. How do you think the helper experience at De Leo Fund can be improved?
16. Any comments you would like to add?
